# Gut Microbiota Impact on Cognitive Function in Humans

**DOI:** 10.3390/nu18030369

**Published:** 2026-01-23

**Authors:** Soghra Bagheri, Ireneusz Ryszkiel, Agata Stanek

**Affiliations:** 1 Medical Biology Research Center, Health Technology Institute, Kermanshah University of Medical Sciences, Kermanshah 6714415185, Iran; 2 Department of Internal Medicine, Metabolic Diseases and Angiology, Faculty of Health Sciences in Katowice, Medical University of Silesia, Ziołowa 45/47, 40-635 Katowice, Poland; ryszkielirek@gmail.com

**Keywords:** gut microbiota, nutrients, intervention, cognitive function, disease

## Abstract

The human gut microbiome and its relationship with both physiological and pathological functions have long intrigued researchers. One of the most fascinating and important areas within this domain is cognitive function. Given that a substantial number of studies, especially interventional ones, have been conducted on animal models, the findings of which are not fully generalizable to humans and may therefore be misinterpreted, the purpose of this study is to synthesize evidence from the most recent human research. Current evidence indicates that the gut microbiota is linked to cognitive function in both healthy and diseased states, with numerous studies suggesting a potential causal relationship between the two. Although the majority of these studies associate changes in cognitive function with differences in the composition of the gut microbiota, some findings also indicate an inverse relationship.

## 1. Introduction

A significant variety of microorganisms, encompassing bacteria, yeasts, and viruses, inhabit numerous regions of the human organism, including the intestines, skin, lungs, oral cavity, and other areas collectively referred to as microbiota. The makeup of the microbiota varies across different regions of the body [1]. An expanding collection of information detailing the architecture and functional abilities of the microbiome across various conditions, along with continuous initiatives aimed at further elucidating microbiome functions and the fundamental processes of host-microbe interactions, is elucidating the role of the microbiome in physiology and in the development of disease [2,3,4].

The gut microbiota is regarded as the most crucial element in sustaining human health [2]. Emerging evidence suggests that gut microbiota composition influences brain function, behavior, and cognition. Cognitive function reflects a lifelong learning process integrating short-term and long-term cognitive mechanisms [5,6].

The aging process is characterized by biological alterations that include cognitive decline and reduced gut microbial diversity. Nevertheless, there is limited understanding regarding the connection between cognitive function and the microbiome as individuals age. A study conducted on healthy individuals aged between 60 and 75 years offers concrete evidence of a correlation between particular bacterial families and various aspects of cognitive function [7].

It should be noted that the number and diversity of gut microbiota strains in both physiological and pathological conditions are strongly influenced by age. Recent evidence indicates that aging is associated with progressive alterations in microbial richness, diversity, and community structure, which may confound or modify disease-associated microbiota signatures and should therefore be considered when interpreting microbiome data in neurodegenerative and metabolic disorders [8].

Using a systematic review and meta-analytic approach, the relationship between the gut microbiome and cognitive function in aging was assessed using studies published between 2013 and 2023. The findings suggested that individuals with cognitive impairment—such as Alzheimer’s disease (AD), Parkinson’s disease (PD), and dementia—exhibited differences in the relative abundance of gut microbiota compared with healthy subjects [9].

Alzheimer’s disease (AD) and Parkinson’s disease (PD) exhibit both overlapping and distinct alterations in the relative abundance of major gut microbiota phyla, including *Firmicutes*, *Bacteroidetes*, *Actinobacteria*, and *Proteobacteria.* Comparative analyses summarized in ref. [8] suggest that AD is more consistently associated with a reduction in *Firmicutes* and *Actinobacteria*, together with a relative increase in *Bacteroidetes* and *Proteobacteria*, whereas PD shows a more pronounced enrichment of *Proteobacteria*, reflecting intestinal inflammation and disease-specific dysbiosis along the gut–brain axis [8]. Moreover, microbiota alterations observed in AD exceed those expected from age-dependent changes alone, supporting the presence of disease-specific dysbiosis rather than purely physiological aging effects [10,11].

While gut microbiota–cognition relationships have been widely studied in cognitively impaired older adults, evidence in cognitively healthy elderly populations remains limited. Healthy aging is associated with reduced abundance of beneficial gut bacteria (e.g., *Bifidobacterium*, *Lactobacillus*) and increased levels of pro-inflammatory taxa, including *Enterobacteriaceae* and *Clostridia*. *Bifidobacterium* and *Lactobacillus,* which tend to decline with age, exert protective effects by supporting gut barrier integrity, producing short-chain fatty acids (SFAs) and other bioactive metabolites, and promoting anti-inflammatory immune responses. Their age-related reduction has been linked to increased intestinal permeability and low-grade systemic inflammation. Conversely, aging is frequently accompanied by an expansion of *Enterobacteriaceae* and certain *Clostridia*, which are associated with enhanced lipopolysaccharide exposure, altered bile acid metabolism, and activation of pro-inflammatory pathways. These changes contribute to the phenomenon of “inflammaging” and may increase susceptibility to metabolic and neurodegenerative disorders, highlighting the functional relevance of age-dependent microbiota alterations [8].

These alterations stand in contrast to those observed in younger and middle-aged subjects and are associated with cognitive status [12]. One of the most significant obstacles in the realm of AD is the delayed identification of the condition [13,14]. Alterations in gut microbiota composition among older adults, even in the absence of a diagnosis for cognitive impairment, might act as preliminary biological indicators for AD or other forms of dementia prior to the onset of mild cognitive impairment (MCI) [12]. This article seeks to offer an overview for readers and researchers who are interested in this domain, focusing on the gut microbiome–cognition axis, as informed by the most recent human studies.

## 2. Sex and Age Impact on the Relationship Between Gut Microbiome and Cognition

Stratification by microbiome profile and correlation analyses with brain age, Mini-Mental State Examination (MMSE) scores, and Clinical Dementia Rating–Sum of Boxes (CDR-SB) scores indicated that brain age may partially mediate the association between gut microbiome dysbiosis and impaired cognitive performance. In that study, the mean MMSE score was 23.23 ± 4.89, consistent with a cohort predominantly composed of individuals with MCI [15].

Limited understanding exists regarding the associations between gut microbiota, metabolic profiles, and young children’s cognitive outcomes. A cross-sectional study involved 452 children aged between six and nine years, during which IQ was assessed, and both fecal microbiota and plasma metabolites were analyzed. The restricted maximum likelihood (REML) analyses indicated that neither microbiota composition nor fecal metabolites had a significant association with subscale or full-scale IQ. However, a notable correlation was found between plasma metabolites and processing speed. The study identifies several microbiota-derived metabolites involved in cognitive function. In particular, SCFAs—including acetate, propionate, and butyrate—are identified as key mediators influencing neuroinflammation, synaptic plasticity, and microglial activation. The study also identifies tryptophan-derived metabolites, such as indole and indole-3-propionic acid, as contributors to neuroprotective signaling and regulation of oxidative stress. In addition, microbial modulation of neurotransmitter-related pathways, including serotonin and gamma-aminobutyric acid (GABA) signaling, is highlighted. Finally, alterations in bile acid metabolism, particularly involving secondary bile acids, are identified as factors affecting mitochondrial function and neuroinflammatory responses, thereby linking gut microbial metabolic activity to cognitive performance [16].

In contrast, other studies have evaluated the connections between gut microbial composition and cognitive measures in middle-aged adults within a well-established population-based study. The findings indicated that β-diversity, an indicator of gut microbial community composition, exhibited significant associations with performance across all cognitive function assessments. Notably, *Barnesiella* and the *Lachnospiraceae FCS020* group exhibited positive associations, while *Sutterella* demonstrated a negative correlation with specific cognitive evaluations [17].

To clarify the sex-specific impacts of gut microbiota on brain function and cognition, healthy young adults underwent various types of MRIs, including structural, perfusion, functional, and diffusion imaging, to evaluate gray matter volume, cerebral blood flow, functional connectivity strength, and white matter integrity. Additionally, fecal samples were collected, and 16S amplicon sequencing was employed to analyze gut microbial diversity. The results underscored sex as a potentially significant factor influencing the relationship between gut microbiota, brain function, and cognition, revealing that gut microbiota may serve as a biomarker-informed and sex-specific target for therapeutic interventions in mental disorders marked by dysregulated behavioral inhibition [18].

In a related study, changes in the gut microbiome and plasma metabolome induced by metformin were found to be associated with cognitive function in males. Specifically, an increase in the *A. muciniphila*/*R. ilealis* ratio was observed in individuals with type 2 diabetes treated with metformin. While this ratio did not show a significant correlation with cognitive test scores in the entire cohort of metformin-treated patients, further analysis by sex revealed a significant positive relationship between the *A. muciniphila*/*R. ilealis* ratio and improved memory scores and performance in men. The increased *Akkermansia muciniphila/Ruminococcus ilealis* ratio observed in patients with type 2 diabetes treated with metformin is likely attributable to the selective modulatory effects of metformin on gut microbiota composition. Metformin accumulates in the intestinal lumen, where it alters bile acid signaling, mucin availability, and the local metabolic environment, thereby favoring the expansion of *A. muciniphila*, a mucin-degrading bacterium associated with improved gut barrier integrity and reduced metabolic inflammation. In contrast, taxa such as *Ruminococcus ilealis* appear less adapted to these metformin-induced conditions and may decrease in relative abundance. Consequently, enrichment of *A. muciniphila* together with suppression of *R. ilealis* results in an increased *A. muciniphila*/*R. ilealis* ratio, reflecting a shift toward a metabolically favorable gut microbiota profile associated with improved insulin sensitivity in metformin-treated individuals [19].

A metagenomics analysis of the gut microbiota among participants categorized by sex (female versus male) and age (<75 versus ≥75 years) revealed that females and younger participants (under 75 years) displayed greater gut microbial diversity, marked by a higher prevalence of *Bifidobacterium* spp. and *Blautia* spp. In contrast, males and older participants (75 years and above) showed elevated levels of *Bacteroides* spp. and *Bacteroidia*, which are linked to inflammation and dysbiosis. Age-related differences between individuals below and above 75 years may be partly explained by age- and sex-related hormonal changes that modulate immune responses, gut microbiota composition, and neurovascular regulation. Declining estrogen levels in women and progressive testosterone reduction in men are associated with increased systemic inflammation and altered gut–brain axis signaling, which may influence cognitive trajectories in advanced age. After the age of 75 years, hormonal levels in both sexes tend to converge at lower ranges, potentially contributing to a more homogeneous pro-inflammatory milieu and increased vulnerability to cognitive decline [20].

Another metagenomics study conducted among centenarians in Hainan revealed significant differences in β-diversity between the sexes, with male centenarians exhibiting a higher level of α-diversity compared to their female counterparts. Thirty-one species were found to be enriched in males, while seven species were identified as enriched in females. Functional analyses indicated that the gut microbiome of males demonstrated greater resilience to oxidative stress (OS). Conversely, among healthy female centenarians, species linked to healthy aging were predominant, whereas those associated with unhealthy aging were comparatively scarce [21].

Sex influences both the number (richness) and composition of gut microbiota strains, with potential consequences for cognitive function. Evidence indicates that women and men differ in microbial diversity profiles across the lifespan, reflecting sex-specific interactions between hormonal status, immune regulation, and aging. In women, particularly after menopause, reductions in microbial richness and diversity have been associated with increased inflammatory susceptibility and may contribute to greater vulnerability to cognitive decline. In men, age-related changes in microbiota composition appear more gradual but are linked to chronic low-grade inflammation and metabolic and vascular risk factors that can negatively affect cognition. Overall, sex acts as an important modifying factor in the relationship between gut microbiota diversity and cognitive outcomes, rather than as an independent determinant [20,21].

The sex-related differences in gut microbiota diversity and composition associated with cognitive aging have been shown in Table 1.

## 3. Gut Microbiome Relationship with Cognitive Impairments in Various Disorders

### 3.1. Mild Cognitive Impairment

Significant differences in gut microbial taxonomic profiles were observed between subjects with and without preclinical AD. Changes in the gut microbiome are associated with the pathological biomarkers Aβ and tau, yet they do not correlate with neurodegeneration biomarkers. This observation implies that changes in the gut microbiome may occur early in the disease progression [22]. Elevated *Bacteroides* prevalence is independently linked to mild cognitive impairment (MCI) in individuals who do not have dementia. Patients with higher levels of *Bacteroides* were more likely to exhibit white matter hyperintensities and elevated voxel-based specific regional analysis system for Alzheimer’s disease (VSRAD) scores, an MRI-derived measure of cortical and hippocampal atrophy [23]. This association represents a statistically significant correlation; however, given the observational design, it should be interpreted as correlational rather than causal.

In a randomized clinical trial of middle-aged and older subjects, microbiome analysis identified *Prevotella ruminicola*, *Bacteroides thetaiotaomicron*, and Bacteroides xylanisolvens as taxa associated with MCI. Baseline analyses revealed a higher abundance of Prevotella in participants with MCI, while probiotic supplementation with Lactobacillus rhamnosus GG was associated with reduced *Prevotella* and *Dehalobacterium* abundance and improved cognitive scores [24]. Another study found that *Ruminococcus gnavus* was more prevalent in patients suffering from cognitive impairment and showed an inverse relationship with the scores obtained from the MMSE) [25]. Research shows diverse outcomes concerning the prevalence of particular bacterial taxa and their links to cognitive functions. Importantly, the correlation between specific bacteria and cognitive abilities may differ when examined across various taxonomic hierarchies, such as phylum compared to family [12].

To date, research on MCI has primarily focused on cross-sectional differences, neglecting longitudinal changes and their potential prognostic implications. A longitudinal study assessing whether taxonomic and functional gut microbiome data from cognitively healthy individuals could predict conversion from normal cognition to MCI over a four-year follow-up found that the best-performing model for identifying MCI converters was based on functional data annotated using Gene Ontology (GO) and included 14 features. This function-based model outperformed the taxonomic model, which incorporated 38 genus-level features, in descriptive accuracy and demonstrated comparable performance to integrated models combining functional, taxonomic, and clinical variables [26]. A case–control investigation involving older adults (ages 55–76), predominantly female, obese, and African American, both with and without MCI, revealed that although the diversity of microbial communities was comparable between the case and control groups, the quantities of certain microbial taxa differed significantly. For instance, *Parabacteroides distasonis* was found to be less abundant in the cases, whereas *Dialister invisus* was more prevalent among them. However, these disparities were no longer evident after controlling for indicators of systemic inflammation and OS. Furthermore, cognitive assessment scores exhibited a positive correlation with the presence of *Akkermansia muciniphila*, a bacterium linked to diminished inflammation [27].

Alterations in gut microbiota composition observed in MCI are increasingly linked to functional metabolic changes that may contribute to early cognitive decline. Enrichment of Gram-negative taxa, particularly *Bacteroides*, is associated with increased production of lipopolysaccharide (LPS), which can translocate across a compromised intestinal barrier and trigger systemic inflammation. LPS-mediated activation of pro-inflammatory cytokines, including tumor necrosis factor alpha (TNF-α), interleukin-1 beta (IL-1β), and interleukin 6 (IL-6), has been implicated in neuroinflammation, white-matter injury, and hippocampal dysfunction in individuals with MCI [23,24]. Concurrently, depletion of SCFAs–producing bacteria leads to reduced availability of butyrate and propionate, metabolites known to maintain intestinal barrier integrity, suppress inflammatory signaling, and support microglial homeostasis. Reduced SCFA levels may therefore facilitate chronic low-grade inflammation and accelerate cognitive impairment. In contrast, higher abundance of *Akkermansia muciniphila*, a mucin-degrading bacterium associated with acetate and propionate production, has been linked to improved cognitive performance, likely through enhancement of gut barrier function and attenuation of inflammatory pathways [27].

The association between mild cognitive impairment (MCI) and increased abundance of *Prevotella ruminicola*, *Bacteroides thetaiotaomicron*, and *Bacteroides xylanisolvens* is likely mediated by shifts in microbial metabolic activity rather than direct neurotoxicity. These taxa are highly efficient degraders of complex carbohydrates and host-derived glycans, favoring metabolic pathways that increase non-butyrate SCFAs production and alter bile acid and lipid metabolism. Such shifts may reduce butyrate-mediated neuroprotective signaling while promoting low-grade systemic inflammation and impaired gut barrier integrity, thereby facilitating neurovascular dysfunction and subtle cognitive decline characteristic of MCI [10,24].

**Figure 1:**
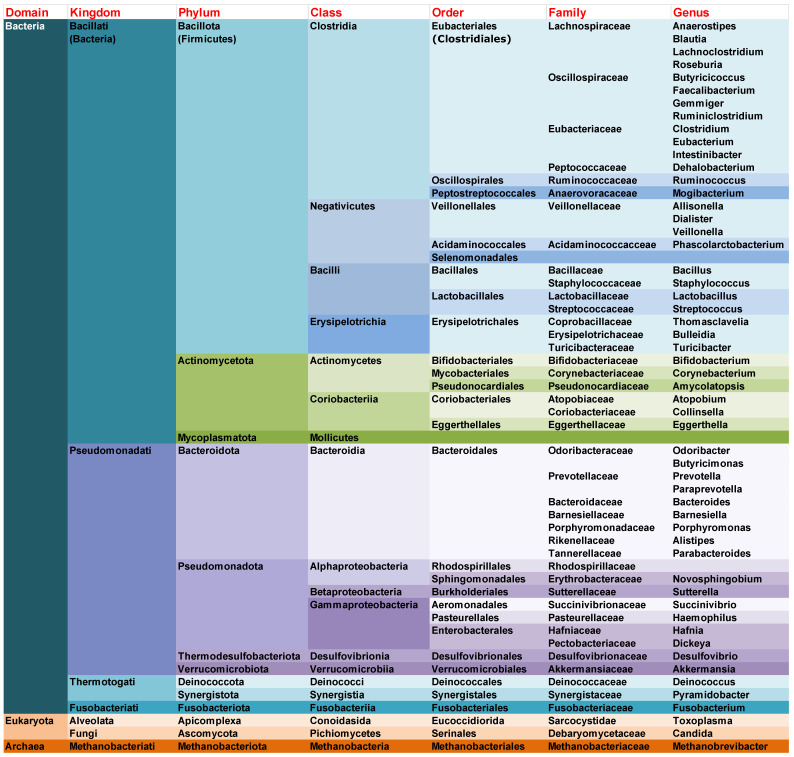
Gut microbiome genera associated with cognitive impairment in different diseases. Background colors indicate major taxonomic groups, with distinct colors representing different domains and phyla, and lighter shades denoting successive lower taxonomic ranks (class, order, and family).

Multiple reports have highlighted the link between gut microbiome and cognitive impairment relationships in different disorders. Figure 1 summarizes the genera that have been associated with cognitive impairment in these diseases, with further details provided later in this section.

### 3.2. Alzheimer’s Disease

Cognitive impairment represents a multifaceted condition, and elderly individuals experiencing cognitive impairment are significantly more prone to developing AD compared to their cognitively healthy counterparts [25]. Limited understanding exists regarding the dysbiosis of the gut microbiome in MCI individuals who may be at an increased risk of AD [26].

Recent multi-omics analyses revealed eight microbial taxa (such as *Staphylococcus* and *Bacillus*) that exhibited a continuous increase in relative abundance throughout the AD continuum, transitioning from healthy controls to individuals with MCI and ultimately to AD. In contrast, two taxa (for instance, *Anaerostipes*) exhibited a steady decrease. Furthermore, 26 fecal metabolites (including Arachidonic, Adrenic, and Lithocholic acids) displayed a progressive rise from healthy controls to MCI and subsequently to AD [10].

Additionally, an investigation involving older adults in Uganda revealed that gut microbiome diversity, assessed through the Chao1 and Shannon indices, was notably diminished in AD patients. Furthermore, genera like *Novosphingobium* and *Staphylococcus* were found to be more prevalent in healthy controls, while *Hafnia-Obesumbacterium* and *Dickeya* were more frequently observed in the AD group [11]. A comprehensive meta-analysis revealed a general reduction in species richness within the gut microbiome of AD patients. Nevertheless, the *Bacteroides* phylum exhibited consistently elevated levels in American cohorts while showing diminished levels in Chinese cohorts. Furthermore, the *Phascolarctobacterium* genus demonstrated a significant rise, albeit exclusively at the MCI phase [28].

An investigation was conducted to examine the associations between the gut microbiome, cognitive impairment, and brain structure using multi-omics data from three independent populations. Participants from the Guangzhou Nutrition and Health Study (GNHS) provided gut microbiome and cognitive assessment data and served as the discovery cohort. Validation analyses were performed in an AD case–control study (replication study 1) and in an additional community-based cohort (replication study 2), both of which used cross-sectional datasets. The study identified protective associations between specific gut microbial genera—*Odoribacter*, *Butyricimonas*, and *Bacteroides*—and cognitive impairment in both the discovery cohort and replication study 1, with findings for *Bacteroides* further confirmed in replication study 2. Moreover, *Odoribacter* was positively correlated with hippocampal volume, a relationship that may be mediated by acetic acid. Notably, greater intra-individual variability in gut microbial composition was observed among subjects with cognitive impairment. In addition, several serum metabolites, as well as inflammation-related metagenomic species and pathways, were identified as being associated with impaired cognitive function [29].

Indeed, earlier meta-analyses revealed considerable heterogeneity among the studies concentrating on AD. Changes in microbial abundance, particularly within certain phyla, were linked to cognitive impairments. Nevertheless, the discrepancies in research outcomes and methodologies underscored the intricate nature of the links between the gut microbiome and cognitive function [9].

Multi-omics analyses across the AD continuum demonstrate that progressive cognitive decline is accompanied not only by taxonomic shifts in the gut microbiota but also by coordinated changes in microbial metabolic outputs. Increased abundance of taxa such as *Staphylococcus* and *Bacillus* from cognitively healthy individuals to MCI and AD parallels elevations in fecal metabolites, including arachidonic acid, adrenic acid, and the secondary bile acid lithocholic acid [25]. These biologically active metabolites directly participate in neuroinflammatory signaling: polyunsaturated fatty acid derivatives such as arachidonic and adrenic acids serve as precursors for pro-inflammatory eicosanoids that activate microglia and promote oxidative stress, whereas lithocholic acid has been linked to mitochondrial dysfunction and neurotoxicity. Concomitantly, AD patients exhibit a progressive loss of beneficial SCFAs–producing taxa. In particular, *Anaerostipes*—a key butyrate-producing genus with anti-inflammatory and neuroprotective properties—shows a steady decrease, together with reductions in *Hafnia–Obesumbacterium* and *Dickeya*. Depletion of *Anaerostipes* likely results in reduced SCFA availability, impairing histone deacetylase inhibition, intestinal barrier integrity, and microglial homeostasis, thereby facilitating neuroinflammation and oxidative stress. This loss of protective SCFA-mediated signaling occurs alongside elevated pro-inflammatory lipid metabolites, indicating a shift from protective to deleterious microbial metabolic pathways during AD progression [10]. In line with these findings, AD patients also display reduced abundance of *Akkermansia muciniphila* and lower levels of propionic acid, SCFAs involved in regulating neuronal mitochondrial dynamics and autophagy via G-protein-coupled receptors GPR41 and GPR43 [30].

At the community level, reduced microbial diversity further amplifies these pathogenic mechanisms. Decreased Chao1 and Shannon diversity indices reported in older adult populations reflect loss of microbial richness—particularly among low-abundance but functionally important taxa such as SCFAs producers—and reduced community evenness with dominance of inflammation-associated genera [11]. These changes imply diminished microbial functional redundancy and metabolic resilience, favoring chronic low-grade inflammation and impaired gut–brain axis signaling that may accelerate neuroinflammatory processes and cognitive decline in AD. Conversely, the lower prevalence of MCI and positive correlations observed with *Odoribacter*, *Butyricimonas*, and *Bacteroides* may be explained by their capacity to generate neuroprotective metabolic signals. *Odoribacter* and *Butyricimonas* are prominent SCFAs producers, particularly of acetate and butyrate, which exert anti-inflammatory effects through inhibition of histone deacetylases, modulation of microglial activation, and suppression of pro-inflammatory cytokine signaling [10]. In parallel, *Bacteroides* species support immune homeostasis and gut barrier integrity, thereby limiting endotoxin translocation and systemic inflammatory signaling that can adversely affect the central nervous system. Integrative analyses further indicate that microbial acetate production—especially from taxa such as *Odoribacter*—is associated with preserved hippocampal volume and better cognitive performance, supporting a neuroprotective role for these metabolites in early cognitive decline [29].

### 3.3. Parkinson’s Disease

A systematic review of the literature provided preliminary evidence linking gut microbiome composition to cognitive function in PD, particularly within the *Bacteroidetes* and *Firmicutes* phyla; however, the associations with specific genera varied across studies.

In PD, cognitive impairment has been associated with reductions in SCFAs-producing bacteria, particularly those generating acetate, propionate, and butyrate. SCFAs exert anti-inflammatory effects by modulating microglial activation, reinforcing gut barrier integrity, and regulating peripheral immune responses. Their depletion may therefore enhance intestinal permeability and facilitate neuroinflammatory signaling pathways implicated in cognitive dysfunction in PD [31]. Additionally, gut microbial expression of tyrosine decarboxylase can metabolize levodopa in the intestine, reducing its bioavailability and potentially contributing indirectly to both motor and cognitive symptom variability [30].

Nevertheless, no statistically significant relationship was identified concerning complications related to motor response [32].

### 3.4. Schizophrenia

In comparison to healthy controls, patients with schizophrenia exhibit higher relative abundances of *Collinsella*, *undefined Ruminococcus*, *Lactobacillus*, *Eubacterium*, *Mogibacterium*, *Desulfovibrio*, *Bulleidia*, *Succinivibrio*, *Corynebacterium*, and *Atopobium*, while the levels of *Faecalibacterium*, *Anaerostipes*, *Turicibacter*, and *Ruminococcus* are found to be lower. Among patients, the positive factor, general factor, and total score of MCCB demonstrate a positive correlation with *Lactobacillus*, *Collinsella*, and *Lactobacillus*, respectively. Conversely, SOD exhibits a negative correlation with *Eubacterium*, *Collinsella*, *Lactobacillus*, *Corynebacterium*, *Bulleidia*, *Mogibacterium*, and *Succinivibrio*, while showing a positive correlation with *Faecalibacterium*, *Ruminococcus*, and the MCCB verbal learning index scores. Additionally, *Faecalibacterium* and *Turicibacter* were positively associated with MCCB visual learning and speed of processing index scores, respectively [33].

A systematic review examining the links between cognition and gut microbiota in subjects with schizophrenia spectrum and mood disorders has pinpointed certain taxa, namely *Haemophilus*, *Bacteroides*, and *Alistipes*, as likely factors that enhance cognitive performance. In contrast, the presence of *Candida albicans*, *Toxoplasma gondii*, *Streptococcus*, and *Deinococcus* was linked to a decline in cognitive assessment outcomes. Furthermore, interventions involving prebiotics and probiotics were found to correlate with improvements in cognitive abilities, especially in the realm of executive functions [34].

More recent research has shown that the prevalence of *Turicibacter* is greater in the group of schizophrenia patients who are classified as overweight or obese, while it is lower in the normal weight patient group when compared to the normal control groups at equivalent BMI levels, respectively. In the overweight/obesity patient group, a rise in *Collinsella* was found to have a significant negative correlation with cognitive function, whereas a reduction in *Clostridium* and *Butyricicoccus* was significantly positively correlated with cognitive function [35].

Cognitive impairment in schizophrenia appears to be closely linked to quantitative alterations in gut microbiota composition and the resulting neuroinflammatory milieu. Schizophrenia-associated dysbiosis is characterized by a reduced abundance of butyrate-producing taxa, including *Faecalibacterium*, *Anaerostipes*, *Clostridium*, and *Butyricicoccus*, together with enrichment of inflammation- and metabolism-associated genera, such as *Veillonella*, *Ruminococcus gnavus*, *Fusobacterium*, *Erysipelatoclostridium*, *Turicibacter*, and *Collinsella*. Butyrate plays a central role in suppressing oxidative stress, maintaining neuronal plasticity, and regulating immune responses through histone deacetylase inhibition and modulation of microglial activity. Consequently, depletion of butyrate-producing bacteria may promote OS and dysregulated cytokine signaling, characterized by elevated pro-inflammatory cytokines (TNF-α, IL-6, IL-1β, and IL-17) and reduced anti-inflammatory cytokines, including IL-10, thereby contributing to microglial activation, synaptic dysfunction, and impaired cognitive performance [35].

In contrast, preservation of cognitive function may be supported by microbiota profiles enriched in butyrate-producing genera, which promote immune homeostasis and synaptic integrity, while limiting the overabundance of taxa such as *Turicibacter* and *Collinsella* may reduce Th17- (T-helper17) driven immune activation and metabolic inflammation. Although *Bifidobacterium* is generally associated with immunoregulatory and anti-inflammatory effects, partly mediated by enhanced IL-10 production, its protective capacity may be insufficient to counterbalance the inflammatory burden in dysbiotic states dominated by pro-inflammatory taxa. Collectively, these findings suggest that microbiota compositions favoring butyrate production and immune regulation may play a protective role in preventing cognitive dysfunction, particularly in schizophrenia patients with metabolic comorbidities, whereas broader dysbiotic shifts rather than single taxa likely drive neuroinflammation-related cognitive impairment [33,34,35].

### 3.5. Depressive Disorder

A systematic review published in 2022 examined associations between the human gut microbiota and cognition, brain function, and mental health outcomes, including stress, anxiety, and depression. Cross-sectional studies identified correlations between microbiota diversity and composition and brain regions relevant to memory and visual processing, while intervention studies aimed at modulating the gut microbiota were associated with improvements in cognitive performance, brain activity, and reductions in anxiety and depression [36].

A recent study involving participants exhibiting a range of cognitive and depressive symptoms revealed that a higher diversity of microbial communities correlates with diminished current cognitive performance across the entire sample, as well as increased depression levels in individuals not receiving antidepressant treatment. Additionally, poor cognitive functioning at present is linked to a reduced relative abundance of *Bifidobacterium*, whereas an increase in GABA degradation is associated with heightened severity of current depressive symptoms. Furthermore, future cognitive decline is related to lower cognitive performance, a decreased relative abundance of *Intestinibacter*, diminished glutamate degradation, and elevated baseline histamine synthesis. The progression of depressive symptoms is predicted by elevated baseline depression and anxiety, lower cognitive function, the presence of diabetes, decreased relative abundance of *Bacteroidota*, and impaired glutamate degradation [37].

Elevated serum concentrations of homocysteine are observed in individuals diagnosed with major depressive disorder. Significant reductions in cognitive performance were observed across six domains: processing speed, working memory, visual learning, reasoning and problem-solving, social cognition, and global cognition. Homocysteine levels were negatively associated with processing speed, social cognition, and total cognitive scores in individuals with major depressive disorder and were also inversely correlated with *Alistipes*, *Ruminococcaceae*, *Tenericutes*, and *Porphyromonas* abundance [38].

Through neuroimaging and microbiome analyses, researchers identified 43 microbial species that exhibited differences in subjects with first-episode major depressive disorder in comparison with the control group. Among these, 11 strains were found to have a significantly increased abundance, while 32 strains showed a significant decrease. The majority of these strains were classified under the *Proteobacteria* phylum, with two strains belonging to *Actinomycetes*. Importantly, the relative abundances of *Amycolatopsis* sp. Hca4, a strain within the *Actinomycetes* phylum that was found to be lower in patients, were shown to modify the links between the functional connectivity of the middle frontal gyrus and the parahippocampal gyrus in relation to working memory. This correlation was observed to vary depending on the abundance of *Amycolatopsis* sp. Hca4. Reduced abundance of *Amycolatopsis* sp. Hca4 may promote brain atrophy by depleting anti-inflammatory polyketides, dysregulating tryptophan-derived metabolites, impairing neurotrophic signaling, and enhancing OS and neuroinflammation [39].

Functional microbial pathways involved in GABA degradation, glutamate metabolism, and histamine synthesis have been associated with both depressive symptom severity and cognitive decline. Increased GABA degradation and impaired glutamate degradation may disrupt excitatory–inhibitory balance within the gut–brain axis, while enhanced histamine synthesis may promote neuroinflammation [38]. Additionally, mediation analyses reveal that circulating free fatty acids (FFAs) partially explain the association between gut microbiota composition and cognitive impairment, suggesting a lipid-driven inflammatory mechanism [40]. Elevated homocysteine levels further reflect metabolic dysregulation interacting with gut microbiota and cognitive outcomes [38].

### 3.6. Vascular Cognitive Impairment

The relationship between the α-diversity index, neuroimaging indicators, and cognitive performance in cases of cerebral small vessel disease (CSVD), both with and without cognitive impairment, was examined. The findings revealed that the Chao1 index was significantly lower in the CSVD subgroups, in contrast to the Shannon index. Furthermore, the Chao1 index exhibited a negative correlation with both the counts of cerebral microbleeds, a neuroimaging feature of CSVD, and the CSVD burden score among patients. In addition, the Chao1 index has been linked to overall cognitive performance, processing speed of information, and language abilities in CSVD patients. Notably, the heightened CSVD burden score acted as a mediator for the impact of reduced Chao1 levels on information processing speed and language function. A reduced Chao1 index with a preserved Shannon index in CSVD reflects selective loss of low-abundance, functionally important microbial taxa, leading to impaired metabolic and immunoregulatory capacity without major disruption of dominant microbial community structure [41].

Individuals suffering from vascular cognitive impairment exhibited a notably higher prevalence of *Bifidobacterium*, *Veillonella*, *Ruminococcus gnavus*, *Fusobacterium*, and *Erysipelatoclostridium*, concomitant with a reduction in the presence of *Collinsella*. Furthermore, *Ruminococcus gnavus* demonstrated a negative correlation with cognitive function in these patients, a relationship that is significantly influenced by cerebral blood flow in both the bilateral hypothalamus and the left amygdala [42].

In vascular cognitive impairment, alterations in gut microbiota diversity and composition have been associated with cerebral small vessel disease burden and reduced cognitive performance. Specific taxa have been shown to influence cognition through modulation of regional cerebral blood flow, suggesting a microbiota–vascular–brain axis. Reduced production of SCFAs and increased LPS-driven inflammation may contribute to endothelial dysfunction, impaired cerebral perfusion, and heightened neuroinflammatory signaling, thereby exacerbating vascular-related cognitive decline [41,42].

### 3.7. Cancer-Related Cognitive Impairment

Approximately 35% of individuals who survive cancer for an extended period encounter persistent cognitive impairments related to their condition, known as cancer-related cognitive impairment (CRCI). CRCI is characterized by chronic inflammation, OS, and alterations in brain structure and function. Gut microbiota dysbiosis in cancer survivors has been associated with reduced abundance of beneficial taxa and altered metabolite profiles. Microbiota-derived SCFAs exert anti-inflammatory effects and support neuronal integrity, whereas their depletion may amplify systemic inflammation and neurotoxicity. In addition, dysregulated tryptophan metabolism and immune-mediated cytokine signaling may further contribute to CRCI pathophysiology [43,44].

The microbiome profiles exhibited notable differences in cancer survivors experiencing cognitive impairment when compared to those who did not have cognitive impairment, and these profiles were also dissimilar to those observed in non-cancer individuals with cognitive impairment. Specific bacterial taxa, such as *Streptococcus thermophilus* and the *Firmicutes bacterium CAG 114*, were found to be significantly diminished in cancer survivors and were strongly correlated with the presence of cognitive impairment [44].

### 3.8. HIV

A cross-sectional investigation was conducted to explore the relationships between gut microbiota and cognitive impairment in women, irrespective of their HIV status. An increased abundance of *Methanobrevibacter*, *Odoribacter*, *Pyramidobacter*, *Eubacterium*, *Ruminococcus*, and *Gemmiger*, along with a decreased abundance of *Veillonella*, was associated with cognitive impairment. Notably, the associations between these microbial taxa and cognitive impairment were stronger in women living with HIV than in HIV-negative participants [45].

Recent studies in individuals living with HIV further demonstrated that those with cognitive impairment exhibited increased abundance of specific bacterial species, including *Desulfovibrio desulfuricans*, *Sutterella wadsworthensis*, and *Streptococcus thermophilus* [46]. In this population, gut microbiota alterations linked to cognitive impairment were also associated with distinct microbial metabolic pathways, particularly enhanced 1,2-propanediol degradation and lipid-related metabolic processes [46]. These pathways may influence host lipid metabolism and inflammatory signaling, thereby contributing to cognitive dysfunction. Moreover, enrichment of pro-inflammatory taxa, especially *Desulfovibrio desulfuricans*, may further promote cytokine-mediated neuroinflammation, particularly in HIV-positive individuals [45,46].

### 3.9. Other Disorders

Across other neurological and systemic disorders, including bipolar disorder, insomnia, and chronic liver disease, cognitive impairment has been associated with microbiota-driven alterations in glucose metabolism, SCFAs production, and inflammatory signaling. SCFAs -producing taxa such as *Faecalibacterium*, *Prevotella*, and *Roseburia* are generally linked to improved cognitive outcomes, whereas enrichment of inflammation-associated genera correlates with greater symptom severity [47,48,49].

The diversity and composition of gut microbiota exhibit significant variations among patients with bipolar disorder who do not have cognitive impairment, those with cognitive impairment, and healthy control subjects. Additionally, bacteria linked to inflammation, such as *Lachnoclostridium* and *Bacteroides*, were found to correlate with the severity of depressive symptoms [47].

Patients suffering from primary insomnia exhibit notable alterations in their gut microbiota, which will aid in clarifying the links between gut microbiota and the cognitive function alterations observed in primary insomnia patients [48].

Microbial populations linked to cognitive impairment were identified solely in the initial phases of liver disease, even though an imbalance of SCFAs was correlated with various stages of the disease. *Streptococcus mutans* and *Allisonella histaminiformans* may act as promising MCI biomarkers [49].

## 4. Mechanisms of Gut Microbiota Related to Cognitive Function

Iron is a critical factor for the proliferation of most bacterial taxa and is also required for myelin production and neurotransmitter biosynthesis. A growing body of clinical evidence highlights the role of the gut microbiota in modulating iron homeostasis and cognitive processes. Data from the Aging Imageomics cohort demonstrated a positive association between circulating ferritin levels and executive function. Conversely, several *Proteobacteria* members—including *Klebsiella pneumoniae*, *Klebsiella michiganensis*, and unclassified *Escherichia*—were inversely correlated with both ferritin concentrations and executive performance. These observations suggest that the gut microbiome may mediate the relationship between iron status and cognitive function through microbially derived metabolites [50].

Among the primary mediators of interactions between hosts and microbes, volatile fatty acids are of significant importance. There are notable differences in the levels of SCFAs in blood and feces, as well as in microbiota composition, between healthy individuals and those diagnosed with AD. AD patients exhibited a reduction in the prevalence of *Akkermansia muciniphila* and a decline in propionic acid levels, both in fecal matter and in blood [30]. Furthermore, a mediation analysis investigating the impact of total FFAs on the relevance of gut microbiota composition and cognitive function in patients suffering from late-life depression revealed that the relationship between reduced relative abundance of *Akkermansia* and cognitive decline was partially mediated by total FFAs which explained 43.0% of the relative effect [40]. *Akkermansia muciniphila*, recognized as a next-generation probiotic, serves as a fundamental regulator of the gut-organ axis across a range of diseases [51]. It plays a central role in maintaining gut barrier integrity and metabolic homeostasis; its reduced abundance is associated with increased intestinal permeability, systemic inflammation, and neuroinflammatory signaling, all of which contribute to cognitive decline [30,51]. Importantly, *Akkermansia* modulates the production of SCFAs particularly propionate, which has been shown to regulate neuronal mitochondrial dynamics and autophagy via activation of G-protein-coupled receptors- (GPR41 and GPR43), thereby reducing microglial activation and supporting neuronal energy homeostasis [30].

Additionally, *Alistipes* and its associated metabolites are pivotal in the pathogenesis of major depressive disorder mediated by the microbiota-gut–brain axis. Multi-omics analyses demonstrate that alterations in *Alistipes* abundance are associated with changes in lipid- and tryptophan-derived metabolites, which modulate neuroimmune signaling, oxidative stress, and brain structure. Specifically, *Alistipes*-related metabolites have been linked to alterations in gray matter volume, white matter integrity, and functional brain connectivity, as well as to cognitive domains such as sustained attention and executive function. Dysregulation of *Alistipes* may therefore promote cognitive decline by enhancing neuroinflammation and disrupting neurotransmitter-related metabolic pathways. Metabolites linked to *Alistipes* have been correlated with a diverse range of brain imaging metrics that encompass gray matter structure, spontaneous brain activity, and white matter integrity. Notably, the functional brain measures were subsequently linked to affective symptoms such as anxiety and anhedonia, as well as cognitive functions like sustained attention in individuals with major depressive disorder [52].

In addition, total FFAs represent a critical metabolic mediator linking gut microbiota composition to cognitive outcomes. Balanced levels of circulating FFAs—particularly unsaturated fatty acids—exert anti-inflammatory and neuroprotective effects, support neuronal membrane integrity, and regulate synaptic plasticity. Mediation analyses in human cohorts demonstrate that total FFAs partially explain the association between gut microbiota alterations, including reduced *Akkermansia* abundance, and cognitive decline, indicating that microbiota-driven lipid metabolism constitutes an important pathway in the gut–brain axis [40].

The investigation into the influence of FFAs on cognitive function mediated by gut microbiota and circulating microRNAs, within a demographic of middle-aged and elderly individuals who are overweight and obese, revealed that cognitive decline was notably present in the overweight and obese groups. Furthermore, it was observed that the intake of dietary saturated fatty acids increased while the consumption of dietary unsaturated fatty acids decreased among individuals in the normal weight category. In the overweight group, the fatty acid C18:3n-3 exhibited beneficial mediating effects on cognitive function. In contrast, C18:3n-6 and hsa-miR-142-5p demonstrated a negative correlation with cognitive performance. Additionally, erythrocyte membrane C23:0, recognized as a reliable marker of dietary fat intake, was found to affect cognitive function through the influence of *Fusobacteriota*, *Proteobacteria*, and plasma hsa-miR-144-3p in individuals classified as obese [53].

**Figure 2:**
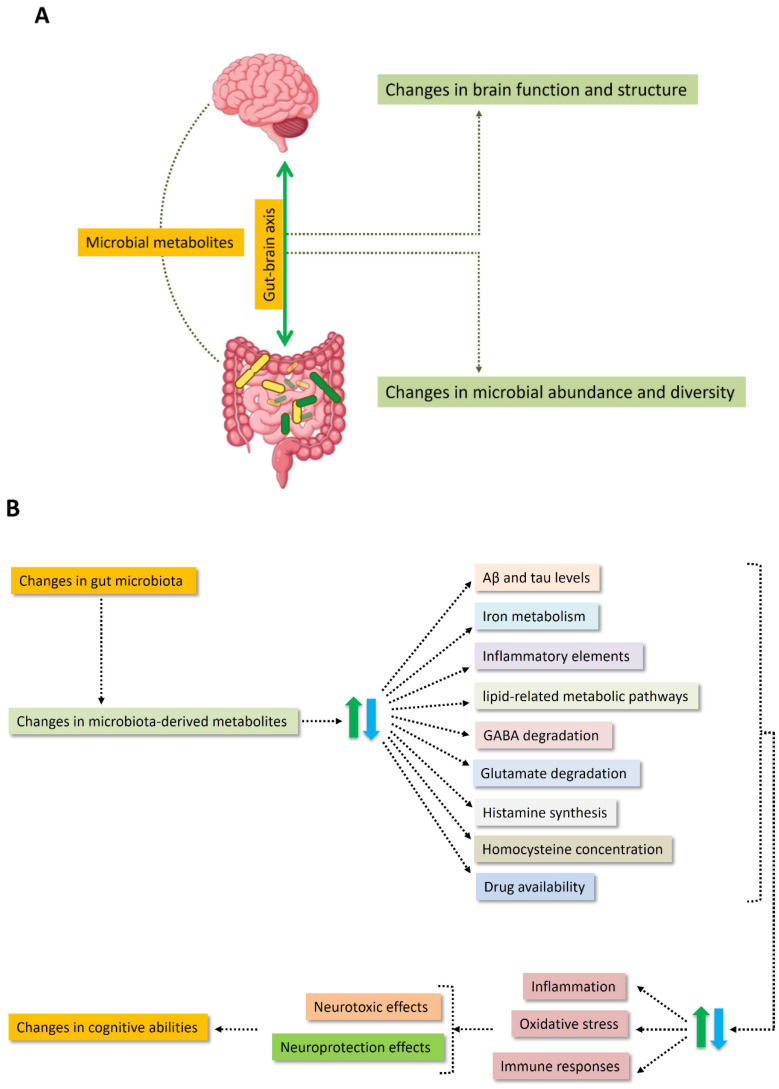
Association between the gut microbiota and cognitive function. (**A**) Changes in brain structure and function are associated with differences in gut microbial composition and diversity, a relationship commonly referred to as the gut–brain axis. Numerous studies support a causal link, highlighting microbial metabolites and the metabolic pathways they modulate as key mediators. (**B**) Proposed mechanisms through which the human gut microbiota may influence cognitive function. Arrows indicate the direction of interactions; dashed arrows denote indirect or modulatory effects, and bidirectional arrows represent reciprocal relationships. Colors are used to distinguish functional domains, including microbiota-related processes, metabolic and molecular pathways, and downstream neurological and cognitive outcomes.

Figure 2 presents an overview of the putative mechanisms by which the human gut microbiota may modulate cognitive function, as reported in the reviewed studies. In general, the gut microbiota exerts a direct influence on host health and cognition through the production of metabolites that regulate multiple biological pathways, including those involved in neuronal function.

## 5. Causal Links Between Gut Microbiota and Cognitive Disorders

Mendelian randomization has gained prominence as a robust analytical method that leverages genetic variation as a natural experiment, thereby reducing confounding and enabling more reliable causal inference [54].

An investigation utilized systematic Mendelian randomization, leveraging data from genome-wide association studies (GWAS), uncovered seven causal links between microbiota or metabolites and cognitive phenotypes. An elevated presence of the order *Clostridiales* was correlated with enhanced cognitive traits, while 1-linoleoyl glycerophosphoethanolamine also showed a positive correlation with cognitive characteristics. Furthermore, seven significant metabolic pathways were identified, including those involved in the metabolism of alpha-linolenic acid and linoleic acid, underscoring the potential influence of omega-3 and omega-6 fatty acids on cognitive health. Additionally, the study revealed two important mediation pathways that connect gut microbiota to cognitive phenotypes via metabolites. Importantly, homostachydrine was identified as a mediator of a portion of the effect of the genus *Turicibacter* on emotion recognition [55].

Another study reported seven positive causal associations between host genetically determined gut microbiota and cognitive performance, including taxa from the class *Clostridia*, order *Clostridiales*, family *Rhodospirillaceae*, and the *Ruminococcus torques* group, as well as the genera *Dialister*, *Paraprevotella*, and *Ruminococcaceae* UCG-003. In contrast, higher abundance of four gut microbiota traits—the genera *Blautia*, *Lachnospiraceae* FCS020 group, *Lachnospiraceae* NK4A136 group, and *Roseburia*—was negatively associated with cognitive performance. In addition, eight positive and six negative associations were identified between genetically predicted metabolite profiles and cognitive outcomes [56].

A Mendelian randomization analysis demonstrated a causal link between chronic kidney disease and cognitive impairment, with the gut microbiome acting as a mediator [57].

A two-sample Mendelian randomization analysis was conducted to evaluate the causal effect of gut microbiota on AD. Seven gut microbial taxa were found to be associated with AD risk. Specifically, the order *Selenomonadales*, family *Pasteurellaceae*, and genus *Methanobrevibacter* were linked to an increased risk of AD, whereas the class *Mollicutes* and the genera *Ruminiclostridium* 9, *Clostridium innocuum* group, and *Eggerthella* were associated with a reduced risk, suggesting a protective effect. No evidence of statistically significant reverse causality between AD and any of these seven gut microbial taxa was observed [58].

Using data from the MiBioGen and FinnGen consortia, multiple Mendelian randomization approaches were employed to comprehensively evaluate the causal associations between 119 gut microbial genera and dementia, while also identifying the most influential taxa. In total, 21 genera were found to exert causal effects on dementia, with *Barnesiella* and *Allisonella* emerging as the principal genera associated with Alzheimer’s disease and all-cause dementia [59].

Previous research has highlighted the important roles of gut microbiota and inflammatory factors in mental and behavioral disorders; however, the magnitude of these effects varies, and the precise causal relationships remain incompletely understood. To further investigate these associations, large-scale genome-wide association study (GWAS) data were analyzed. The findings revealed causal links, with inflammatory factors playing a mediating role in the pathway connecting gut microbiota to these disorders [54].

In contrast to studies highlighting the influence of gut microbiota on brain function, there are also findings indicating that alterations in cognitive abilities can result in parallel modifications in microbiota compositions. For instance, elderly patients experiencing MCI who participated in a randomized controlled trial focused on mindful awareness practices showed improvements in cognitive impairment that were associated with changes in their gut bacterial profiles. This implies that signals emanating from various regions of the brain may directly or indirectly regulate the populations of certain gut microbes [60].

## 6. Nutrients Beneficial to the Human Gut Microbiota

One of the most effective ways to modulate the gut microbiota is through targeted dietary choices, which profoundly shape its structure, composition, metabolic activity, and overall function [61]. A large-scale analysis involving 21,561 subjects from five multinational cohorts demonstrated that gut microbial profiles clearly distinguished major dietary patterns, including omnivorous, vegetarian, and vegan diets. Red meat intake was identified as a key determinant of the omnivorous microbiome, with characteristic taxa—such as *Ruminococcus torques*, *Bilophila wadsworthia*, and *Alistipes putredinis*—showing inverse associations with cardiometabolic health. In contrast, microbial signatures typical of vegan diets were linked to more favorable cardiometabolic markers and were also more prevalent among omnivores with higher consumption of plant-based foods [62].

Additionally, a recent cross-sectional study reported that higher total dairy and milk consumption, combined with lower cheese intake, was associated with increased gut microbial alpha diversity. Greater intake of dairy products and milk correlated with a higher relative abundance of *Faecalibacterium*, while increased milk consumption was specifically associated with elevated levels of *Akkermansia*. Conversely, higher consumption of total dairy and cheese was linked to a reduced abundance of *Bacteroides* [63]. Notably, *Faecalibacterium* is a major producer of butyrate and other short-chain fatty acids through dietary fiber fermentation and is widely recognized for its anti-inflammatory effects [64].

Overall, diets rich in prebiotics, fermented foods, and plant-derived bioactive compounds—such as polyphenols and flavonoids—promote greater microbial diversity and stability [65].

## 7. Nutritional Interventions and Potential Therapeutic Approaches for Cognitive Changes Mediated by Gut Microbiota Alteration

Research shows that modifications to gut microbiota can influence cognitive behavior. A summary of the reviewed studies was presented in Table 2. In a placebo-controlled, double-blind, randomized controlled trial involving 36 pairs of twins aged 60 and above, each pair was block-randomized to receive either a placebo or a daily prebiotic for a duration of 12 weeks. The administration of the prebiotic supplement led to alterations in the gut microbiome (for instance, an increase in the relative abundance of *Bifidobacterium*) and enhancements in cognitive performance [66].

Numerous research studies have demonstrated the positive impact of an anti-inflammatory diet on the modulation of gut microbiota [67]. Anti-inflammatory diets have been linked to a greater presence of advantageous microbes, and a particular gut microbial composition has been connected to cognitive function. Within the fecal microbiota, the genus *Haemophilus* exhibited a negative correlation with MoCA and VFT scores. In contrast, *Holdemanella* and *Porphyromonas* demonstrated a positive association with MoCA and VFT scores [68].

**Table 2:** Nutritional strategies and possible therapeutic methods aimed at modifying microbiota to enhance cognitive performance.

Intervention	Gut Microbiota Changes	Outcomes	Reference
Daily prebiotic	Alterations in the gut microbiome	Enhancements in cognitive performance	[66]
Anti-inflammatory diet	Greater presence of advantageous microbes	Better MoCA and VFT scores	[68]
Nuts	Increase in gut microbial diversity	Favorable changes in global cognitive function	[69]
		Slower decline in attention	
Asafoetida	Decrease in *Firmicutes*/*Bacteroidetes*	Enhanced cognitive function	[70]
	Enhance in α-diversity	Better sleep quality	
Low FODMAP diet	Reduction in *Actinobacteria*, *Firmicutes*, *Lactobacilli*, and *Bifidobacteria*	Enhancement of all neurological symptoms	[71]
	Increase in α-diversity Decline in *Firmicutes*/*Bacteroidetes* ratio		
Probiotics	Lower relative abundance of gut bacteria associated with inflammation	Improvement in mental flexibility tests	[72]
		Decrease in stress scores	
Supplementation with *Lactobacillus*	Greater alterations in abundance of beneficial microorganisms within the LL group	Enhancements in cognitive function assessments	[73]
Multidomain intervention with nutritional supplements	Enhance in β-diversity	Improvement in RBANS scores	[74]
Laparoscopic sleeve gastrectomy	Increase in abundance and diversity of gut microbiota	Enhancements in cognitive functions	[75]
Combination of fiber-rich diet with rope skipping	Enhancement in the evenness of the microbial community	Reductions in both prospective and retrospective memory impairments	[76]
Aerobic exercise training	Alteration of gut microbiota	Improve working memory and inhibitory control	[77]
Exercise training	Increase in the levels of *Akkermansia muciniphila* and *Faecalibacterium*	Enhancements in cognitive abilities	[78]
	Decrease in *Lactobacillus* abundance		
Exercise interventions	Increase in gut microbiome diversity	Enhancements in psychomotor speed	[79]
		Improvement in executive function	

According to the results of a prospective study involving older adults who are at risk of cognitive decline, those who consumed between 3 and 7 servings of nuts per week exhibited a significantly slower deterioration in global cognitive function during the follow-up period when compared to participants who consumed one or fewer servings per week. This category of nut consumption was also linked to an increase in gut microbial diversity. Thirteen taxa were found to be associated with nut consumption, with ten showing a positive correlation, including *Lachnospiraceae UCG-004*, which was additionally linked to favorable changes in global cognitive function and a slower decline in attention [69].

Asafoetida played a crucial role in altering the gut microbiota by decreasing the ratio of *Firmicutes* to *Bacteroidetes*, enhancing α-diversity, promoting beneficial genera such as *Bacteroides* and *Prevotella*, and diminishing harmful species like *Escherichia* and *Clostridia*. The corresponding modulation resulted in enhanced cognitive function and better sleep quality [70].

The clinical management of a young patient with autism spectrum disorder complicated by epilepsy and metabolic disturbances, who showed limited response to standard therapies, included the use of ketogenic and low-FODMAP dietary interventions. The ketogenic diet was associated with a reduction in the relative abundance of *Firmicutes*, *Bacteroidetes*, and *Proteobacteria*. Subsequent transition to a low-FODMAP diet led to marked improvement in neurological, gastrointestinal, and metabolic symptoms and was well tolerated. Gut microbiota analysis following the dietary intervention demonstrated decreased levels of *Actinobacteria*, *Firmicutes*, *Lactobacillus*, and *Bifidobacterium*. In addition, α-diversity increased consistently, while the *Firmicutes*/*Bacteroidetes* ratio declined, suggesting an attenuation of fermentative dysbiosis [71].

In a randomized, double-blind, placebo-controlled multicenter trial, probiotics containing *Bifidobacterium bifidum* BGN4 and *Bifidobacterium longum* BORI were administered for 12 weeks to community-dwelling older adults to evaluate their effects on cognitive function and mood. Participants receiving probiotics exhibited a significantly lower relative abundance of inflammation-associated gut bacteria, along with greater improvements in mental flexibility and stress-related scores compared with the placebo group. Moreover, serum brain-derived neurotrophic factor (BDNF) levels increased significantly in the probiotic group but not in the placebo group. Notably, gut microbial taxa most affected by probiotic supplementation, particularly *Eubacterium* and *Clostridiales*, demonstrated a statistically significant inverse association with serum BDNF levels only in the probiotic-treated group [72].

Supplementation with *Lactobacillus delbrueckii subsp. lactis CKDB001* (LL) led to significantly greater enhancements in cognitive function assessments compared to the placebo group. Taxonomic and metabolomic analyses of fecal samples revealed notably greater alterations in the relative abundance of beneficial microorganisms within the LL group, with the most significant changes observed at the family (*Lactobacillaceae*, *Bifidobacteriaceae*) and genus (*Lactobacillus*) levels. Furthermore, the LL group demonstrated significantly elevated fecal concentrations of indole-derived metabolites, such as 5-hydroxyindole-3-acetic acid, indole-3-lactic acid, and indole-3-glycol [73].

A systematic review of pertinent randomized controlled trials involving patients with AD and MCI who primarily received *Lactobacillus* and *Bifidobacterium* strains indicated several positive outcomes regarding cognitive function, modifications in gut microbiota composition, and favorable impacts on metabolic biomarkers. Nevertheless, the inconsistency in microbiota evaluation among the studies constrains the ability to interpret the findings [80].

In a randomized controlled trial, individuals with MCI or mild dementia who were amyloid-PET positive were allocated to one of three groups: group A received a multidomain intervention combined with nutritional supplementation, group B received nutritional supplementation alone, and the third group served as a control. After an 8-week intervention period, the total index score of the Repeatable Battery for the Assessment of Neuropsychological Status (RBANS) improved significantly in group A compared with both group B and the control group. Furthermore, post-intervention analysis revealed greater gut microbiome β-diversity in group A than in controls, along with an increased relative abundance of *Bifidobacterium* among participants in group A [75].

Laparoscopic sleeve gastrectomy resulted in marked weight loss accompanied by improvements in cognitive performance, along with increased gut microbiota diversity and relative abundance. Following bariatric surgery, the intestinal microbiome showed a higher proportion of *Bacteroidetes* and *Fusobacteria*, while the relative abundance of *Firmicutes*, *Proteobacteria*, and *Actinobacteria* decreased. Postoperatively, plasma concentrations of IL-1β and TNF-α were significantly reduced, whereas IL-4 levels increased significantly. Montreal Cognitive Assessment (MoCA) scores were significantly associated with IL-4, TNF-α, and IL-1β. In addition, *Firmicutes* demonstrated a positive correlation with TNF-α, *Fusobacteria* showed a negative correlation with IL-1β, and *Bacteroidetes* was inversely associated with IL-4 [81]. Consistent with these findings, a systematic review reported that bariatric surgery exerts a significant beneficial impact on cognitive function in subjects with obesity [74].

Several bacterial taxa belonging to the *Proteobacteria* (including *Escherichia coli*) and *Verrucomicrobia* (notably *Akkermansia muciniphila*) phyla were positively associated with metformin treatment, whereas species from the *Firmicutes* phylum—such as *Romboutsia timonensis* and *Romboutsia ilealis*—were negatively associated [19].

A randomized, parallel-group controlled trial in young adults showed that both a fiber-rich diet (FD) and FD combined with rope skipping (RS) were associated with reductions in prospective and retrospective memory impairments. With respect to the gut microbiota, α-diversity did not increase; however, microbial community evenness improved following the FD and RS interventions. Additionally, the relative abundance of the phylum *Firmicutes* and the genera *Faecalibacterium* and *Eubacterium coprostanoligenes* group increased significantly in the RS group, while the *NK4A214* group showed a significant increase in the FD group. Notably, in the RS group, improvements in microbial evenness were positively correlated with enhanced retrospective memory performance [76].

While the effects of dietary fiber on gut microbiota composition and cognitive function have been well documented in adults, the interplay between fiber intake, the gut microbiome, and cognitive outcomes in children remains poorly understood. A non-randomized trial conducted with Thai school-aged children revealed that the consumption of Sinlek rice did not significantly change the abundance of gut microbiota or the cognitive performance of these children. However, the study did identify a correlation between age and variations in both gut microbiota profiles and cognitive outcomes, with older children performing better than their younger counterparts on cognitive assessments [82].

Aerobic exercise training has been shown to enhance working memory and inhibitory control in individuals with methamphetamine dependence. Evidence from gut microbiota analyses suggests that exercise-induced modifications of the gut microbial composition and associated metabolic pathways contribute to improvements in cognitive function in this population [77]. In postmenopausal women with type 2 diabetes mellitus, exercise training resulted in a marked increase in *Akkermansia muciniphila* and *Faecalibacterium* abundance, alongside a reduction in *Lactobacillus*. These microbial changes were accompanied by improvements in HDL cholesterol levels, cognitive performance, and overall physical fitness, with increases in *Akkermansia* significantly associated with enhanced HDL levels and cognitive outcomes [78]. Additionally, a randomized controlled trial in individuals living with HIV demonstrated that exercise interventions produced modest but significant gains in psychomotor speed and executive function, reductions in body mass index, improved physical fitness, and increased gut microbiome diversity. Higher baseline phylogenetic diversity of the gut microbiome was also associated with superior fitness and cognitive performance [79].

## 8. Conclusions

A number of studies have reported relationships between the gut microbiota and cognitive function, as well as cognitive impairments in various diseases. This relationship is considered causal, with microbial metabolites and metabolic pathways playing a mediating role. The diversity of microbial species linked to cognitive function suggests that species composition and functions are more important than taxonomic classifications. Factors like age, gender, and health conditions also influence the impact of microbiota on cognitive function.

Diet represents a key modifiable lifestyle factor with the potential to enhance cognitive function. Although nutritional research has traditionally emphasized brain development during early life, increasing attention is now being directed toward the influence of diet on age-related cognitive decline. Clarifying the role of dietary patterns and specific nutrients in cognitive health may facilitate the development of novel strategies for the prevention, management, or treatment of age-related cognitive disorders and for improving quality of life in older adults. Evidence suggests that low-fat dietary patterns may confer protection against cognitive decline, whereas the cognitive effects of high-protein diets remain inconclusive. Micronutrients, including B vitamins, iron, and polyphenols, play important roles in maintaining cognitive function. Moreover, adherence to dietary patterns such as the Mediterranean, Nordic, DASH, and MIND diets has been linked to a lower risk of cognitive decline and dementia [83].

It has been demonstrated that diet and nutrients may impact the gut microbiome. Ongoing research aims to further understand how dietary changes can modify the gut microbiome to potentially prevent or treat cognitive disorders.

## Figures and Tables

**Table 1:** Sex-related differences in gut microbiota diversity and composition associated with cognitive aging.

Study	Population	Female-Associated Gut Microbiota Characteristics	Male-Associated Gut Microbiota Characteristics
Hong et al., 2025 [20]	Adults stratified by sex and age (<75 vs. ≥75 years)	Higher α-diversity; increased abundance of *Bifidobacterium* spp. and *Blautia* spp.; microbiota profile associated with healthier aging	Higher abundance of *Bacteroides* spp. and class *Bacteroidia*; microbiota profile linked to inflammation and dysbiosis
Luan et al., 2024 [21]	Centenarians (Hainan cohort)	*Prevotella copri*, *Prevotella stercorea*, *Eubacterium rectale*, *Roseburia inulinivorans*, *Coprococcus comes*, *Dorea longicatena* (total *7 species* enriched) Enrichment of SCFAs and taxa linked to healthier aging profiles; *Dorea longicatena* represents an exception, as it has been associated with poorer cognitive performance despite female enrichment	*Lactobacillus gasseri*, *Lactobacillus oris*, *Lactobacillus salivarius*, *Eggerthella lenta*, *Clostridium hathewayi*, *Clostridium difficile*, *Anaerotruncus colihominis*, *Actinomyces viscosus*, multiple taxa belonging to the family *Lachnospiraceae* (total 31 enriched); Higher overall microbial diversity; enrichment of taxa associated with metabolic flexibility and oxidative stress adaptation; several taxa linked to inflammaging and immune modulation

Abbreviations: SCFAs, short-chain fatty acids.

## Data Availability

No new data were created or analyzed in this study. Data sharing is not applicable to this article.

## Abbreviations

The following abbreviations are used in this manuscript:

AD	Alzheimer’s disease
BDNF	Brain-derived neurotrophic factor
CDR-SB	Clinical Dementia Rating Sum of Box
CRCI	Cancer-related cognitive impairment
CSVD	Cerebral small vessel disease
FD	Fiber-rich diet
FFAs	Free Fatty Acids
FODMAP	Fermentable oligosaccharides, disaccharides, monosaccharides and polyols
GABA	Gamma-aminobutyric acid
GNHS	Guangzhou Nutrition and Health Study
GO	Gene Ontology
GWAS	Genome-wide association studies
HDL	High-density lipoprotein
HIV	Human immunodeficiency virus
IL-1β	Interleukin-1 beta
IL-4	Interleukin 4
IL-6	Interleukin 6
IL-10	Interleukin 10
LPS	Lipopolysaccharide
MCCB	MATRICS Consensus Cognitive Battery
MCI	Mild cognitive impairment
MiaGB	Microbiome in Aging Gut and Brain
MMSE	Mini-Mental State Examination
MoCA	Montreal Cognitive Assessment
OS	Oxidative stress
PD	Parkinson’s disease
PET	Positron emission tomography
RBANS	Repeatable Battery for the Assessment of Neuropsychological Status
REML	Restricted maximum likelihood
RS	Rope skipping
SCFAs	Short-chain fatty acids
Th17	T-helper 17
TNF-α	Tumor necrosis factor alpha
VFT	Verbal Fluency Test
VSRAD	Voxel-based specific regional analysis system for AD
